# Systemic infection with insect-specific viruses does not affect *Plasmodium* sporozoite formation in *Anophele*s mosquitoes

**DOI:** 10.1371/journal.pntd.0013848

**Published:** 2025-12-26

**Authors:** Michelle Schinkel, Gijs J. Overheul, Ellen Ploeger, Geert-Jan van Gemert, Sandra Junglen, Pascal Miesen, Teun Bousema, Ronald P. van Rij

**Affiliations:** 1 Department of Medical Microbiology, Radboud University Medical Center, Nijmegen, the Netherlands; 2 Institute of Virology, Charité-Universitätsmedizin Berlin, Corporate Member of Freie Universität Berlin, Humboldt-Universität zu Berlin, and Berlin Institute of Health, Berlin, Germany; International Atomic Energy Agency, AUSTRIA

## Abstract

Malaria remains a leading cause of morbidity and mortality in large parts of the world. Resistance threatens current preventative and curative strategies, highlighting the need for novel complementary strategies. The opportunities that the mosquito virome may offer to reduce pathogen transmission have not been systematically explored for malaria control. In this study, we assessed whether insect-specific viruses affect *Plasmodium* development in mosquitoes. A panel of 15 viruses was tested for *in vitro* and *in vivo* replication in anopheline cells and mosquitoes. From this panel, the RNA viruses Flock House virus, Wallerfield virus, Agua Salud alphavirus, Herbert herbevirus and Gouléako goukovirus and the dsDNA virus invertebrate iridescent virus 6 efficiently replicated in two *Anopheles gambiae* cell lines and were further assessed in *in vivo* experiments. Intrathoracic injection of these viruses in *Anopheles stephensi* mosquitoes resulted in efficient viral RNA replication for Herbert herbevirus, Agua Salud alphavirus and Gouléako goukovirus and high levels of infectious viral particle production for invertebrate iridescent virus 6. In contrast, Wallerfield virus showed poor replication, whereas Flock House virus replicated efficiently but caused high mosquito mortality. Subsequently, we performed *in vivo* coinfections of *Plasmodium falciparum* with the four efficiently replicating insect-specific viruses that were not associated with high mosquito mortality. Sporozoite formation and *Plasmodium* infection rates did not differ between virus-infected mosquitoes and non-infected controls. While none of the tested viruses significantly affected *Plasmodium* development, our study identified multiple viruses that efficiently infect *Anopheles stephensi* mosquitoes, providing a useful resource to study *Anopheles* immunity. Future studies may address whether native *Anopheles*-specific viruses affect *Plasmodium* parasite transmission.

## Author summary

Malaria continues to be a major health issue in many endemic countries, and current prevention and treatment methods are not fully effective. This study explores a new way to combat malaria transmission using insect-specific viruses to reduce *Plasmodium* development in mosquitoes. A panel of 15 different insect viruses was tested for replication in mosquito cells and adult female mosquitoes. Four efficiently replicating viruses were analysed in co-infection experiments, where mosquitoes were first infected with the viruses and subsequently exposed to malaria parasites in an infectious bloodmeal. No significant differences were observed in parasite development, suggesting that persistent infection with these viruses does not affect malaria transmission. Whether other, *Anopheles* specific viruses affect *Plasmodium* parasite transmission remains to be investigated.

## Introduction

Malaria, caused by *Plasmodium* parasites, is a leading cause of morbidity and mortality in many endemic countries. Despite significant progress in lowering disease burden, there were still 263 million malaria cases and 597,000 deaths worldwide in 2023 [1]. Malaria also imposes a substantial economic burden globally, with annual governmental spending exceeding USD 4.3 billion for prevention and treatment [2], while profoundly affecting economic growth in endemic countries. Two partially effective malaria vaccines have recently become available for use in endemic regions, which are likely to have a large beneficial public health impact, yet provide incomplete protection from infection and clinical disease [3–5]. Other methods, like effective antimalarial drugs, insecticide-impregnated bednets and indoor-spraying, have greatly contributed to reduced malaria burden, however, the rise of insecticide resistance in mosquitoes and drug resistance in parasites threatens their effectiveness [6]. Novel, complementary intervention strategies are thus considered essential to achieve malaria elimination.

Parasite transmission to mosquitoes starts with the uptake of male and female gametocytes that activate in the mosquito midgut and, as a result of fusion and zygote formation, develop into a motile ookinete. These ookinetes penetrate the mosquito midgut wall to form an oocyst attached to the gut basal lamina that enlarges over time to release sporozoites into the mosquito hemocoel, which then invade the salivary glands [7–11]. At each step of parasite development in the mosquito, there is a considerable loss in parasite numbers and these losses are at least in part attributable to the mosquito immune system [12]. Upon parasite recognition inside the blood meal, hemocytes are recruited and production of reactive oxygen and nitrogen species increases [13,14]. Subsequently, upon ookinete perturbation of the midgut membrane, the parasite is recognized by pattern recognition receptors that activate the Toll/Rel1 [15], IMD/Rel2 [15–18] and Jak-Stat [19,20] pathways, leading to the production of antimicrobial peptides (AMPs) like Cecropins, Defensins and Gambicins that can act as antiplasmodial factors [9,21,22]. Concurrently, recruited hemocytes initiate processes to lyse or encapsulate the forming oocyst via complement and melanization cascades [23,24].

Symbiotic microbes may form the basis for innovative intervention strategies to prevent malaria transmission. For example, colonization of *Anopheles* mosquitoes with the *Delftia tsuruharerensis* bacterium reduced oocyst formation by approximately 75% [25]. Alternatively, genetically modified *Serratia* or *Pantoea* bacteria are being explored to drive the expression of antimalarial effector proteins in the mosquito midgut to reduce *Plasmodium* infection rates [26,27]. Antimalarial immune mechanisms may also be activated by biological agents like bacteria or viruses [28], and prior administration or infection with such an agent may prime the mosquito immune system to more efficiently reduce subsequent *Plasmodium* infection [29].

*Anopheles* mosquitoes can be infected with a range of insect-specific viruses, which infect mosquitoes but are not transmitted to vertebrates. However, the consequences of systemic virus infection on pathogen transmission have thus far not been studied [29–31]. Evidence from other mosquito species suggests that insect-specific viruses may reduce replication and transmission potential of vector-borne viruses by a mechanism called superinfection exclusion, where prior infection prevents or reduces infection with a second, related virus [32–35]. For example, systemic infection of *Aedes aegypti* with Eilat virus, an insect-specific Alphavirus, reduced infection rates at early time points and delayed dissemination of the arthropod-borne chikungunya virus [32]. These observations suggest that systemic infection with insect-specific viruses may affect replication of coinfecting pathogens, possible by priming immune responses of the mosquito.

In this study, we analysed whether systemic infection with insect-specific viruses affects *Plasmodium* development in *Anopheles* mosquitoes. We screened a panel of available viruses for *in vitro* replication in two *Anopheles gambiae* cell lines and, for efficiently replicating viruses, *in vivo* in *Anopheles stephensi* mosquitoes. Next, we established an *in vivo* virus-parasite coinfection model in *An. stephensi* to assess viral interference with *Plasmodium* infection. While the selected viruses did not affect sporozoite formation and infectivity rates, the characterized viruses and virus-parasite coinfection model may be useful tools for future studies of *Anopheles* immunity.

## Results & discussion

### Screen of insect-specific viruses for replication in *Anopheles* cell lines

In this study, we set out to identify insect-specific viruses that affect replication of the *Plasmodium* parasite in *Anopheles* mosquitoes. While prior knowledge of activation or suppression of mosquito immunity would be useful for selecting candidate viruses, *Anopheles* responses to viral infection had only been studied in the context of infection with the arthropod-borne O’nyong nyong virus [28]. We therefore took an agnostic approach and screened a diverse panel of viruses, available in the laboratory, for *in vitro* replication in the 4a-3B [36,37] and mos55 [38] hemocyte-like cell lines, derived from neonate *An. gambiae* larvae. The screen comprised viruses belonging to 12 different viral genera with diverse genome characteristics and replication strategies. Specifically, we included the positive-sense RNA viruses cell fusing agent virus (CFAV), Kamiti river virus (KRV), cricket paralysis virus (CrPV), Flock House virus (FHV), Wallerfield virus (WALV), Piura virus (PIUV), goutanap virus (GANV), Agua Salud alphavirus (ASALV) and Cavally virus (CAVV), the segmented, negative-sense viruses Ferak orthoferavirus (FERV), Jonchet orthojonvirus (JONV), Gouléako goukovirus (GOLV) and Herbert herbevirus (HEBV), the bisegmented dsRNA virus Culex Y virus (CYV), and the dsDNA virus invertebrate iridescent virus 6 (IIV6) (Table 1). These viruses had been isolated from diverse hosts, including *Aedes* and *Culex* (mosquitoes), *Teleogryllus* (crickets), *Costelytra* (beetles) and *Galleria* (moths) (Table 1). Only GOLV was previously detected in wild-caught *Anopheles* mosquitoes as well as in *Culex* and *Uranotaenia* spp. mosquitoes [39]. For the other viruses, replication in *Anopheles* had not been previously demonstrated.

**Table 1:** Viruses used in this study.

Genus	Family	Virus	Abbr.	Host species^a^	GenBank accession	Genome	Organization	Envelope	Ref.^a^
Alphamesonivirus	Mesoniviridae	Cavally virus	CAVV	Aedes harrisoni	GCA_000891195.1	(+) RNA	non-segmented	yes	[40]
**Alphanodavirus**	**Nodaviridae**	**Flock House virus**	**FHV**	**Costelytra zealandica**	**GCA_000854385.1**	**(+) RNA**	**segmented**	**no**	**[41]**
**Alphavirus**	**Togaviridae**	**Agua Salud alphavirus**	**ASALV**	**Culex declarator**	**GCA_031192855.1**	**(+) RNA**	**non-segmented**	**yes**	**[42]**
Cripavirus	Dicistroviridae	cricket paralysis virus	CrPV	Teleogryllus oceanicus and T. commodus	GCA_000853145.1	(+) RNA	non-segmented	no	[43]
Entomobirnavirus	Birnaviridae	Culex Y virus	CYV	Culex pipiens complex	GCA_031851955.1	dsRNA	segmented	no	[44]
Insect-specific orthoflavivirus^b^	Flaviviridae	cell fusing agent virus	CFAV	Aedes aegypti (cells)	GCA_000862225.1	(+) RNA	non-segmented	yes	[45]
Insect-specific orthoflavivirus^b^	Flaviviridae	Kamiti River virus	KRV	Aedes macintoshi	GCA_000851165.1	(+) RNA	non-segmented	yes	[46]
**Goukovirus**	**Phenuiviridae**	**Gouléako goukovirus**	**GOLV**	**Culex nebulosus**	**GCA_002814655.1**	**(-) RNA**	**segmented**	**yes**	**[39]**
**Herbevirus**	**Peribunyaviridae**	**Herbert herbevirus**	**HEBV**	**Culex nebulosus or Culex decens**	**GCA_002831165.1**	**(-) RNA**	**segmented**	**yes**	**[47]**
**Iridovirus**	**Iridoviridae**	**invertebrate iridescent virus 6**	**IIV6**	**Chilo suppressalis**	**GCA_000838105.1**	**dsDNA**	**non-segmented**	**yes**	**[48]**
Nelorpivirus	Negevirus	Piura virus	PIUV	Culex sp.	GCA_002024735.1	(+) RNA	non-segmented	yes	[49]
Orthoferavirus	Feraviridae	Ferak orthoferavirus	FERV	Culex nebulosus or Culex decens	GCA_002814355.1	(-) RNA	segmented	yes	[50]
Orthoferavirus	Jonviridae	Jonchet orthojonvirus	JONV	Culex decens	GCA_002831065.1	(-) RNA	segmented	yes	[50]
Sandewavirus	Negevirus	goutanap virus	GANV	Culex nebulosus	GCA_000926435.1	(+) RNA	non-segmented	yes	[51]
**Sandewavirus**	**Negevirus**	**Wallerfield virus**	**WALV**	**Culex declarator**	**GCA_000916075.1**	**(+) RNA**	**non-segmented**	**yes**	**[52]**

Viruses in bold were analysed in *in vivo* experiments.

^a^ Organism from which the virus was first isolated and the associated reference.

^b^ Not formally classified yet.

**Fig 1:**
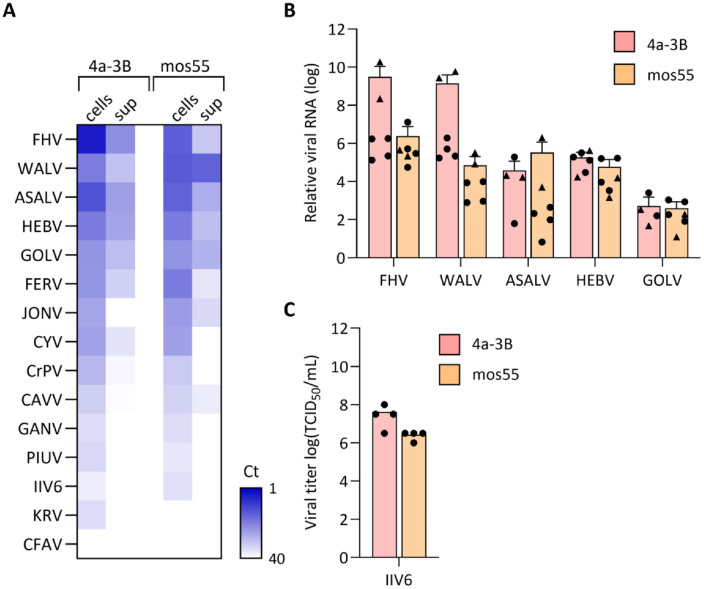
*In vitro* replication of insect-specific viruses in *Anopheles gambiae* cell lines. **(A)** Heat map showing Ct values of RT-qPCR for viral RNA in *An. gambiae* 4a-3B (left) and mos55 cells (right) and their culture supernatant at 3 days post infection with the indicated viruses (MOI = 0.01). Data are ordered from top to bottom according to viral RNA levels. IIV6 is a DNA virus and no viral RNA is expected in the supernatant. Data are the mean Ct of duplicate wells in a single experiment. **(B)** Relative quantification of the top-five viruses from panel A compared to input viral RNA in cells harvested directly after inoculation. Individual data points are shown, with the triangles corresponding to the initial screen and the circles to two additional experiments with duplicate wells, bars indicating the mean +SD. **(C)** IIV6 infectious titers in culture supernatant of 4a-3B and mos55 cells. Data are from two experiments containing duplicate wells, individual data points are shown with bars indicating the mean.

Both 4a-3B and mos55 cells were inoculated with each of the insect-specific viruses at 0.01 infectious viral particles per cell (multiplicity of infection, MOI), and intracellular and extracellular viral RNA levels were assessed at three days post infection using RT-qPCR. The amount of viral RNA after three days of infection in cells and/or supernatant varied per virus and cell line (Fig 1A). For six viruses (FERV, GOLV, HEBV, ASALV, WALV and FHV) we detected viral RNA in both the cells and supernatant, indicating active and spreading viral infection. All replicating viruses could use both cell lines as a host, with a general trend of lower replication in the mos55 cell line. For some viruses (KRV, PIUV, GANV, CAVV, CrPV, CYV and JONV), viral RNA was readily detected in the cellular fraction, but limited viral RNA was found in the supernatant, suggesting inefficient viral RNA packaging, virion assembly or release. CFAV was the only virus for which viral RNA was undetectable both in cells and the supernatant after three days, indicating absent replication. For only one of the viruses, IIV6, visible cytopathic effect (CPE) with cell death and syncytia formation was observed in both 4a-3B and mos55 cells (S1 Fig). As expected for a DNA virus, no viral RNA was detected in the supernatant, however the clear CPE and syncytia suggest that the virus was actively replicating. We repeated infections with the top-five replicating viruses defined by Ct values < 20 for cells and Ct values < 30 for supernatant (FHV, WALV, ASALV, HEBV and GOLV), as well as IIV6 in two additional experiments. At three days post-infection, viral RNA levels increased by 10^2^ to 10^10^-fold relative to input for FHV, WALV, ASALV, HEBV and GOLV (Fig 1B) and IIV6 reached high titers of 10^6^ to 10^8^ TCID_50_/mL (Fig 1C), confirming efficient replication.

### Viral infection does not activate NF-κB pathways in the *An. gambiae* cell lines

Antimalarial defences are regulated by the NF-κB-like Toll/Rel1 and IMD/Rel2 pathways, activation of which leads to the production of AMPs such as Defensin 1 (DEF1, AGAP011294), Cecropin 1 (CEC1, AGAP000693), and Gambicin 1 (GAM1, AGAP008645) [9,21,22]. We therefore analysed expression of these AMP genes upon viral infection with the top-five replicating viruses and IIV6 at three days post infection by RT-qPCR. As a positive control, heat-inactivated *E. coli* was used to activate mos55 and 4a-3B cells. As expected, all three AMP genes were upregulated approximately 10-fold upon *E. coli* stimulation in both cell lines compared to non-stimulated control. In contrast, no significant upregulation of either AMP was observed upon viral infection of *Anopheles* cells (S2 Fig). These data indicate that both the 4a-3B and mos55 cell lines are immune competent, as observed previously [36,37,53], but do not activate AMP expression upon viral infection.

### ASALV, FHV, GOLV, HEBV, and IIV6 efficiently replicate in *Anopheles* mosquitoes *in vivo*

**Fig 2:**
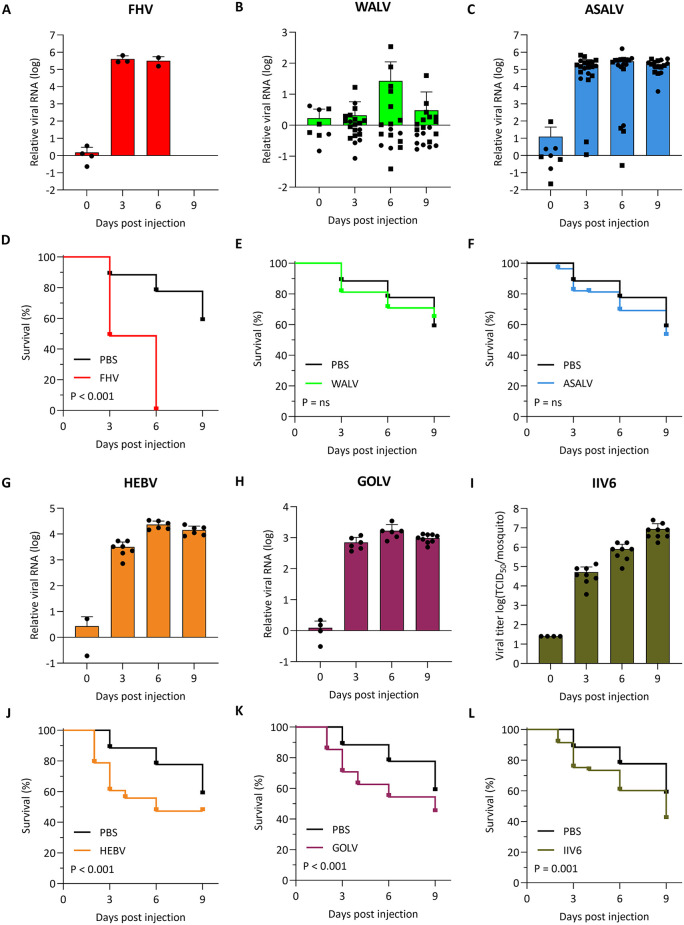
*In vivo* replication of insect-specific viruses in *Anopheles stephensi* mosquitoes. **(A–C, G** and **H)** Relative viral RNA quantification and **(I)** viral titers for the indicated viruses at 0, 3, 6 or 9 days after intrathoracic inoculation (150 PFU) of adult, female *An. stephensi*. Data points represent individual mosquitoes in a single experiment for FHV, HEBV, GOLV and IIV6 and two experiments for WALV and ASALV (distinguished by circles and squares). All FHV infected mosquitoes were dead at 6 days post injection, viral RNA at that timepoint was quantified in two dead mosquitoes. Bars represent means +SD. **(D–F, J–L)** Kaplan-Meier survival curves of virus infected mosquitoes and PBS injected control mosquitoes. For clarity, the same curve of the PBS control is shown in all panels. *P* values from Log-rank Mantel Cox test are indicated. ns, not significant.

We selected the top-five replicating RNA viruses (FHV, WALV, ASALV, HEBV, GOLV) and the DNA virus IIV6 to investigate *in vivo* viral replication in *An. stephensi*, a traditional malaria vector in India and invasive in urban settings in Africa [54]. To this end, we inoculated *An. stephensi* mosquitoes by intrathoracic injection and harvested mosquitoes for quantification of viral RNA (FHV, WALV, ASALV, HEBV and GOLV) or infectious titers (IIV6) at three, six, and nine days post injection. Consistent with the *in vitro* experiments (Fig 1), FHV, ASALV, HEBV, and GOLV replicated efficiently in *An. stephensi*, reaching 10^3^ to 10^5^-fold higher viral RNA levels compared to the inoculum control, achieving 100% infection rates, and plateauing at three or six days post-injection (Fig 2A, 2C, 2G, and 2H). Similarly, IIV6 efficiently replicated *in vivo*, reaching 100% infection rates and peak titers at nine days post infection, which were approximately 10^5^-fold higher than the inoculum (Fig 2I). Among the viruses tested, WALV was the only one that did not seem to efficiently replicate in *An. stephensi* (Fig 2B), which was somewhat surprising given efficient *in vitro* replication (Fig 1). While low levels of viral RNA seemed to accumulate in some mosquitoes, especially at six days post injection, viral RNA levels remained below input levels in over half the mosquitoes.

In parallel to assessing viral replication, we monitored mosquito survival (Fig 2D–2F and 2J–2L–2L). FHV infection was highly lethal, inducing 100% mortality between day 3 and 6 (Fig 2D). In striking contrast, ASALV did not significantly affect survival, even though it accumulated to similar RNA levels as FHV (Fig 2F). Survival of HEBV, GOLV and IIV6 infected mosquitoes differed significantly from mock infected controls (PBS injection, *P* < 0.001, Log rank Mantel Cox test), yet approximately half the mosquitoes survived at 9 days post infection. Consistent with its inefficient replication, WALV did not induce mortality (Fig 2E).

### Insect-specific viruses do not interfere with *Plasmodium* infections

**Fig 3:**
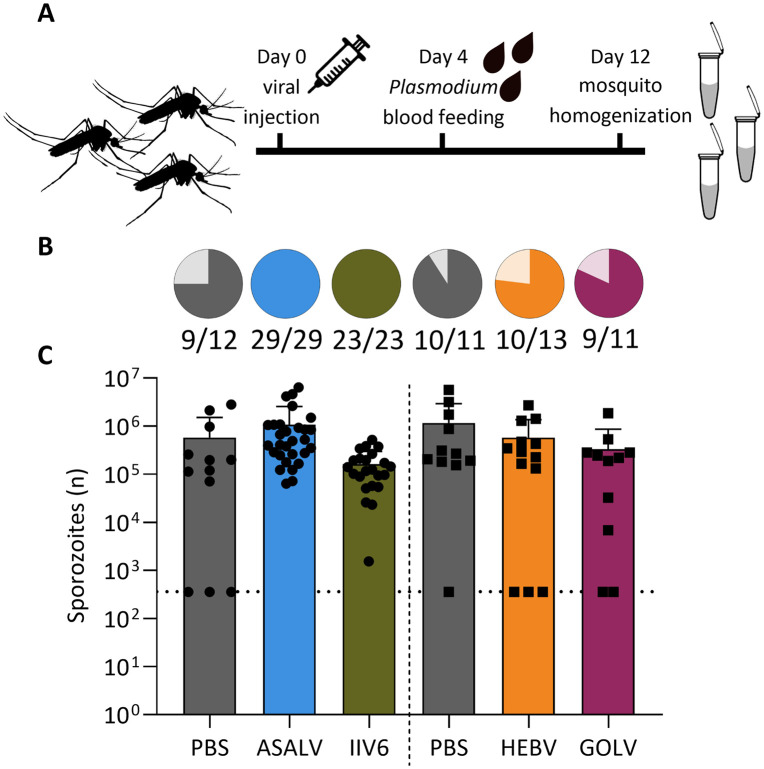
*Plasmodium* infection levels upon viral coinfection in *Anopheles stephensi* mosquitoes. **(A)** Outline of the experiment. Mosquitoes were intrathorically inoculated with virus or PBS at day 0. At day 4, the injected mosquitoes were offered a *Plasmodium* containing blood meal after which engorged mosquitoes were selected. At day 12, the mosquitoes were lysed for analysis. **(B)** Infection rates and **(C)** sporozoite levels in virus infected mosquitoes at 8 days after infectious blood meal (day 12 of the experiment), quantified using RT-qPCR. Data from individual mosquitoes are shown as symbols with bars representing means +SD. Data from one experiment including ASALV, IIV6 and PBS control are shown as circles and data from a second experiment including HEBV, GOLV and PBS control are shown as squares (the vertical dashed line separates the two experiments). The dotted horizontal line shows the sporozoite detection limit. *Plasmodium* sporozoite levels were compared to each internal PBS control with Kruskal-Wallis tests and infection rates were assessed with Chi-squared tests. No significant differences were found. The mosquito icon was made by Mariana Ruiz Villareal (CC0; bioicons.com/icons/cc-0/Animals/Mariana_RuizVillareal/Mosquito_gender.svg), the syringe icon by Derek Croote (CC0; bioicons.com/icons/cc-0/Lab_apparatus/Derek-Croote/simple-syringe-cartoon.svg), and the tube icon by Andi Wilson (CC-BY 4.0; bioicons.com/icons/cc-by-4.0/Microbiology/Andi-Wilson/Eppendorf_dilutionseries.svg).

Given efficient replication and potential for immune activation *in vivo*, we next assessed whether systemic infection with insect-specific viruses affects *Plasmodium* infection in *An. stephensi* mosquitoes. To establish this *in vivo* virus-parasite coinfection model, we first tested how intrathoracic injection affects blood feeding rates by counting fully fed mosquitoes when a blood meal is offered at 24, 48, 72 and 96 hours after PBS injection. Blood feeding rates dropped by approximately 50% at 24 and 48 h after intrathoracic injection compared to non-injected mosquitoes, but feeding rates returned to normal levels (86%) at 96 h (S3 Fig), coinciding with viral growth entering the log phase or plateau (Fig 2). We therefore decided to provide an *P. falciparum* blood meal four days after intrathoracic virus inoculation and to harvest mosquitoes eight days thereafter (Fig 3A). This time point captures the mid-stage of parasite development in the mosquito midgut, just prior to oocyst rupture and sporozoite migration, when sporozoites in the mature oocysts can be detected by molecular methods [10,55].

We analysed sporozoite formation and viral nucleic acid levels in the virus-parasite coinfection model using ASALV, HEBV, GOLV and IIV6, viruses that efficiently replicated, but did not induce overt mortality (Fig 2). We found that *Plasmodium* infection rates did not differ between virus infected mosquitoes and mock infected mosquitoes (Fig 3B). Moreover, for all virus infected mosquitoes, the number of sporozoites in individual mosquitoes did not differ significantly from that in mock infected mosquitoes (Fig 3C). Finally, linear regression analyses revealed no significant correlations between sporozoite numbers and viral RNA or DNA levels (S4 Fig).

**Fig 4:**
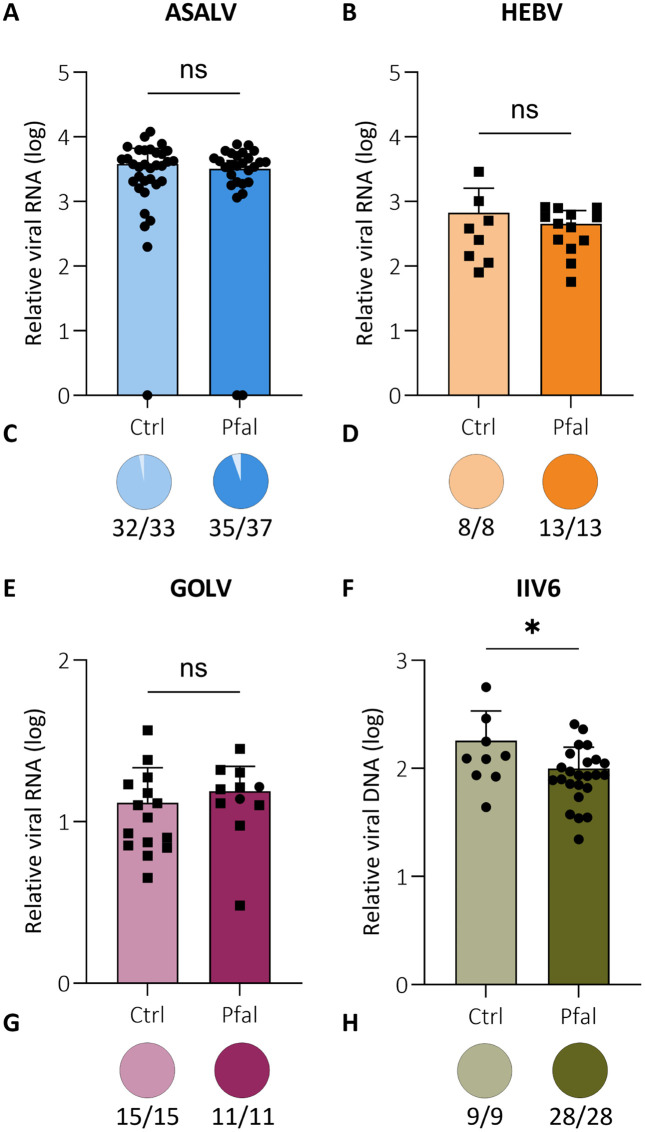
Viral nucleic acid levels upon *Plasmodium* coinfection in *Anopheles stephensi* mosquitoes. Relative viral nucleic acid levels and infection rates of **(A,C)** ASALV, **(B,D)** HEBV, **(E,G)** GOLV and **(F,H)** IIV6 infected mosquitoes at 7 days after a control (ctrl) or *Plasmodium falciparum* (Pfal) infected blood meal. Symbols indicate data for individual mosquitoes, with bars representing means +SD. The circled and square data points represent two experiments using different batches of blood. Negative samples are plotted along the x-axis, but were not used to calculate the mean. Statistical significance was assessed using unpaired *t*-tests for viral RNA and Chi-squared tests for infection rates. (*P < 0.05; ns, not significant).

To further explore potential virus-parasite interactions, we analyzed whether parasite infection affected virus replication. However, viral RNA levels and infection rates for ASALV, HEBV and GOLV did not significantly differ upon subsequent *Plasmodium* infection compared to blood fed but non-parasite infected controls (Fig 4A–4C). Interestingly, viral DNA levels for IIV6 were slightly lower in parasite infected mosquitoes compared to control mosquitoes (*P* = 0.0381, Fig 4D), suggesting that *Plasmodium* infection creates an environment that is less supportive of IIV6 replication. Taken together, our results suggest that systemic infection with these insect-specific viruses does not affect sporozoite formation in *Anopheles* mosquitoes.

## Conclusion

In this study, we assessed whether systemic infection with insect-specific viruses interferes with *Plasmodium* development in mosquitoes, potentially affecting malaria transmission. Starting from a screen of 15 viruses, we identified a large number of viruses that replicated in two *An. gambiae* cell lines. We confirmed efficient replication of a subset of these viruses, FHV, WALV, ASALV, HEBV, GOLV and IIV6, in *An. stephensi* mosquitoes *in vivo*. We found no evidence that any of these viruses affected *P. falciparum* sporozoite levels or infection rates.

Our study has several limitations that should be taken into consideration. First, we used *An. stephensi* as our *in vivo* vector model, which may not fully represent the infection and transmission dynamics of *An. gambiae* or other primary malaria vectors in endemic regions. Second, viral infections were established via intrathoracic injection to obtain a consistent, near 100% viral infection rate. This inoculation route bypasses the natural route of infection and may alter virus-mosquito interactions. In future studies, it will be of interest to test other inoculation routes, especially oral inoculation, which may result in infection of the midgut, the site of *Plasmodium* entry into the mosquito body. Third, with the exception of GOLV, none of the viruses tested have thus far been found to naturally infect *Anopheles* mosquitoes [30]. It will therefore be of considerable interest to analyse native *Anopheles* viruses [30,31] for *Plasmodium* interference, both as a potential biocontrol measure and to improve understanding of natural malaria transmission. Fourth, sporozoite loads were assessed at a time point when sporozoites still reside in mature oocysts, before rupture and salivary gland invasion. These numbers may not accurately reflect numbers of sporozoites expelled from the salivary glands, which would be a more direct readout for transmission potential. As sporozoites still need to migrate to and invade the salivary glands, it remains possible that sporozoite numbers are affected by systemic viral infection at later stages of the infection. Despite these limitations, the tools established in this study will be useful in further dissecting antiviral immunity and the potential interactions between viruses, parasites and mosquitoes.

## Methods

### Cell culture

The *An. gambiae* mos55 cell line, kindly provided by Jason L. Rasgon (The Pennsylvania State University) [38], the *An. gambiae* 4a-3B cell line (BEI resources, MRA-919) [36,37], and the *Drosophila melanogaster* S2 cell line [56] were cultured in Schneider’s medium (Gibco) supplemented with 10% heat inactivated fetal calf serum (FCS) (Sigma-Aldrich), 50 U/mL penicillin and 50 µg/mL streptomycin (pen-strep, Sigma-Aldrich). The *Aedes albopictus* C6/36 cell line (ATCC CRL-1660) [57]) was cultured in Leibovitz’s L-15 medium (Gibco) supplemented with 10% heat inactivated FCS, 2% tryptose phosphate broth solution (Sigma-Aldrich), 1x MEM non-essential amino acids (Gibco), 50 U/mL penicillin and 50 µg/mL streptomycin. All insect cells were incubated at 28°C without CO_2_ supplementation.

### Viruses

ASALV, CAVV, CYV, FERV, GANV, GOLV, HEBV, JONV, PIUV, and WALV were available from previous studies (for references, see Table 1). CFAV (Rio Piedras02 strain) and KRV (SR-75 strain) were provided by the European Virus Archive. CrPV and FHV were kindly provided by Carla Saleh (Pasteur institute) and the IIV6 isolate was kindly provided by Just Vlak (Wageningen University). Viral stocks of CYV, CrPV, FHV and IIV6 were titrated by end-point dilution assays on S2 cells, as described previously [58,59], and the viral stocks of ASALV, CAVV, CFAV, GANV, KRV, PIUV and WALV were titrated on C6/36 cells. Briefly, 1x10^6^ cells/mL were seeded in 96-well flat-bottom plates, incubated overnight and inoculated with a 10-fold serial dilution of virus samples in quadruplicate. After an incubation of 5–7 days and/or when cytopathic effects (CPE) did not change for two consecutive days, viral titers were calculated as TCID_50_/mL using the Reed and Muench method [60]. The TCID_50_/mL values were then converted to plaque forming units (PFU) using the Poisson distribution [61]. FERV, GOLV, HEBV and JONV do not show strong CPE. Relative genome copies in these viral stocks were therefore quantified by RT-qPCR on a dilution series of viral RNA and used to estimate infectious titers using a La Crosse encephalitis virus stock with a known titer as a reference.

### Mosquito culture

*Anopheles stephensi* mosquitoes (Sind-Kasur strain) [62] were reared at 30°C and 80% humidity with a reverse day–night cycle (12 h:12 h). Adult mosquitoes were maintained at 27°C and 80% humidity under a day-night cycle (12 h:12 h), with *ad libitum* access to a 10% sucrose solution.

### *In vitro* viral infection

4a-3B and mos55 cells were seeded at a density of 3 x 10^6^ cells/mL in a 24-well plate, incubated overnight, and inoculated at a multiplicity of infection (MOI, the number of infectious viral particles per cell [61]) of 0.01 for each virus in complete cell culture medium. Cells and supernatant were harvested separately at 3 days post infection in 1 mL RNA-Solv Reagent (Omega Bio-tek). Virus inoculum and cells harvested directly after inoculation were used as input controls. *In vitro* viral infections were performed in three independent experiments with duplicate wells.

### Bacterial stimulation

*Escherichia coli* (XL10-Gold, Agilent Technologies) was cultured overnight on LB-agar plates. Individual colonies were grown in liquid culture of 50 mL LB medium in a shaking incubator to an OD_600_ of 1.0 (corresponding to approximately 8.0 x 10^8^ cells/mL [63]). The bacteria were heat inactivated at 95°C for 15 minutes and stored in aliquots at -80°C until use. For stimulation experiments, 4a-3B and mos55 cells were seeded at a density of 3 x 10^6^ cells/mL in a 24-well plate, incubated overnight, and 25 μL of a suspension of heat inactivated *E. coli* (OD_600_ of 1.0) was added to the medium. Cells were harvested in 1 mL RNA-Solv Reagent (Omega Bio-tek) directly after inoculation (negative control) and after three days.

### *In vivo* viral infection

Three to five-day-old female mosquitoes were used for all experiments. Mosquitoes were infected with the insect-specific viruses via intrathoracic injection of 150 PFUs in a volume of 50 nL or with 50 nL PBS as a mock infection, using a Nanoject III microinjector (Drummond). Viral replication was assessed at different time points via (RT-)qPCR or end-point dilution assays at 3, 6 and 9 days. Whole mosquitoes were homogenized in 100 µL PBS using a Precellys 24 homogenizer (Bertin technologies) and stored at -80°C until titrations (IIV6) or lysed in RNA-Solv Reagent (Omega Bio-tek) and stored at -20°C until RT-qPCR (FHV, WALV, ASALV, HEBV, GOLV). This was done in one experiment for FHV, HEBV, GOLV and IIV6 and two independent experiments for WALV and ASALV.

### Virus-parasite coinfection

*Plasmodium falciparum* parasites (strain NF135) [64] were cultured in an automated incubator as previously described [65]. Mosquitoes were blood fed four days after viral inoculation. Briefly, cups containing 37–56 mosquitoes were exposed for 15 min to feed on human venous blood collected in LiHep BD Vacutainer tubes (Sanquin, Nijmegen, The Netherlands), with or without *Plasmodium* parasites, using the Hemotek feeding system (PS6120) with 1 mL reservoirs (FU1–1) (Discovery Workshops, UK) and Parafilm M as feeding membrane [66]. Afterwards, mosquitoes were anesthetized on a CO_2_ pad, blood feeding rates were assessed by visual inspection, and unfed and partially fed mosquitoes were removed. Viral replication was assessed at different time points via (RT-)qPCR or end-point dilution assays at 12 days post inoculation and *Plasmodium* sporozoite levels were assessed at 8 days post *Plasmodium* infection via (RT-)qPCR. Infection rates were expressed as a percentage or the ratio of the number of infected individuals per total sample population.

### Nucleic acid isolation, reverse transcription and qPCR

Cells and homogenized mosquitoes were lysed in RNA-Solv Reagent (Omega Bio-tek) and total RNA was isolated according to the manufacturer’s instructions. During the coinfection experiments, total nucleic acids (NA) were isolated using the MagNA Pure 96 DNA and Viral NA small volume kit (MagNAPure 96, Roche) according to the manufacturer’s protocol. For RNA quantifications, samples were treated with DNase I (Invitrogen) before cDNA synthesis using the TaqMan Reverse Transcription Reagents kit (Applied Biosystems) according to the manufacturers’ protocol. Viral and parasite RNA or DNA levels and host gene expression were measured by qPCR using the GoTaq qPCR Master Mix (Promega) according to the manufacturer’s instructions on a Light Cycler 480 (Roche). RPL5 was used as the housekeeping gene for *Anopheles* samples. Primer sequences are provided in S1 Table. Threshold values (Ct values) were used to calculate the relative viral RNA levels using the 2^-ΔCt^ method [67]. For sporozoite counts, a standard curve was derived from a serial dilution of a previously determined in-house sample, and relative counts of the experimental samples were determined using the fitted curve. Negative samples in bar graphs were plotted as values at the detection limit, but these values were not used to calculate the mean.

### Statistical analyses

Survival curves were generated using the Kaplan-Meier nonparametric estimation from censored data, and statistical significance was determined using Log rank Mantel Cox tests [68]. Differences in AMP expression were examined with one-way ANOVA for multiple comparisons and sporozoite counts with Kruskal-Wallis tests, comparing each sample group against its internal control. Viral RNA or DNA infection levels were analysed using unpaired *t*-tests on log-transformed data. Infection rates were compared using Chi-squared tests, and correlations were analysed using the Pearson correlation method. All statistical tests were performed using GraphPad Prism 10.

## Supporting information

S1 Fig IIV6 infection causes cytopathic effect in *Anopheles gambiae* cells. Microscopy image of uninfected (left) and IIV6 infected (right) 4a-3B and mos55 cells.
(TIF)

S2 Fig AMP induction upon insect-specific virus infection of *Anopheles gambiae* cell lines. Relative mRNA expression of the AMP genes (A, D) *CEC1*, (B, E) *DEF1* and (C, F) *GAM1* in (A–C) 4a-3B cells and (D–F) mos55 cells at 3 days after viral infection (FHV, WALV, ASALV, HEBV, GOLV, IIV6) at an MOI of 0.01 or after stimulation with heat inactivated (HI) *E. coli*. Expression was measured by RT-qPCR and expressed relative to expression in non-stimulated cells. Individual data points shown are as circles. Bars indicate the mean of three experiments with duplicate wells for all *E. coli* stimulations and three experiments with one individual well for the viruses and non-stimulated control. Statistical significance of each stimulated or infected group was compared to the non-stimulated control using one-way ANOVA (*P < 0.05; **P < 0.005).
(TIF)

S3 Fig Blood feeding rates of *Anopheles stephensi* mosquitoes after intrathoracic injection. Bars represent the ratio of the percentage of blood-fed PBS injected mosquitoes over the percentage of blood-fed non-injected control mosquitoes with 20–27 mosquitoes within each group in one experiment.
(TIF)

S4 Fig No correlation between *Plasmodium* and viral infection levels in coinfected mosquitoes. Each datapoint represents one virus and *Plasmodium* coinfected mosquito, with the *Plasmodium* (*COX1*) Ct value on the x-axis and corresponding viral Ct value on the y-axis for (A) ASALV, (B) HEBV, (C) GOLV and (D) IIV6. Linear regression lines were fitted, and Pearson R^2^ and *P* values are indicated.
(TIF)

S1 Table Sequences of primers used in this study. (PDF)
