# Supplementary figures and images for: Systemic infection with insect-specific viruses does not affect Plasmodium sporozoite formation in Anopheles mosquitoes
Source: PLoS Negl Trop Dis. 2025 Dec 26;19(12):e0013848. doi: 10.1371/journal.pntd.0013848 (PMC12768362; doi:10.1371/journal.pntd.0013848)

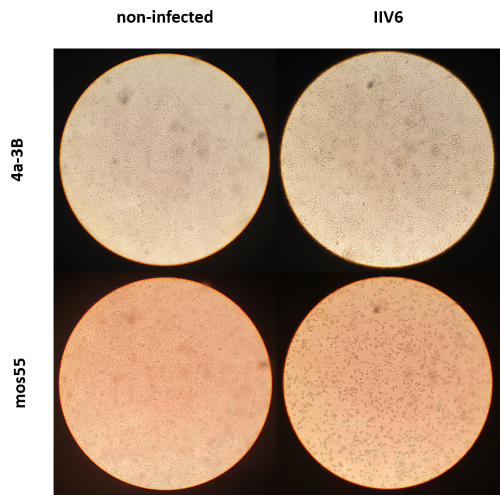

Supplement: S1 Fig — Microscopy image of uninfected (left) and IIV6 infected (right) 4a-3B and mos55 cells. (TIF) [file pntd.0013848.s001.tif]

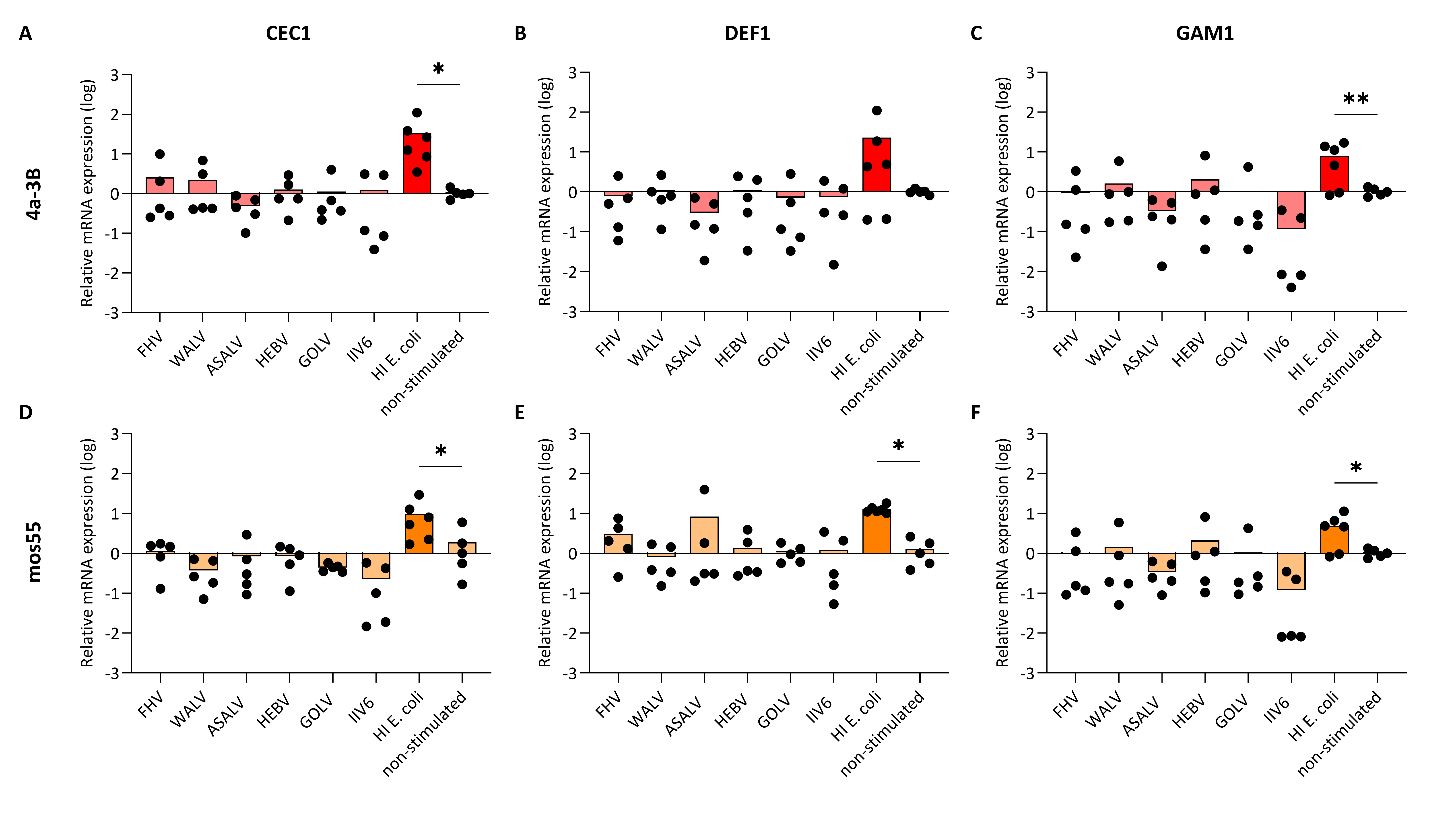

Supplement: S2 Fig — Relative mRNA expression of the AMP genes (A, D) CEC1, (B, E) DEF1 and (C, F) GAM1 in (A–C) 4a-3B cells and (D–F) mos55 cells at 3 days after viral infection (FHV, WALV, ASALV, HEBV, GOLV, IIV6) at an MOI of 0.01 or after stimulation with heat inactivated (HI) E. coli. Expression was measured by RT-qPCR and expressed relative to expression in non-stimulated cells. Individual data points shown are as circles. Bars indicate the mean of three experiments with duplicate wells for all E. coli stimulations and three experiments with one individual well for the viruses and non-stimulated control. Statistical significance of each stimulated or infected group was compared to the non-stimulated control using one-way ANOVA (*P < 0.05; **P < 0.005). (TIF) [file pntd.0013848.s002.tif]

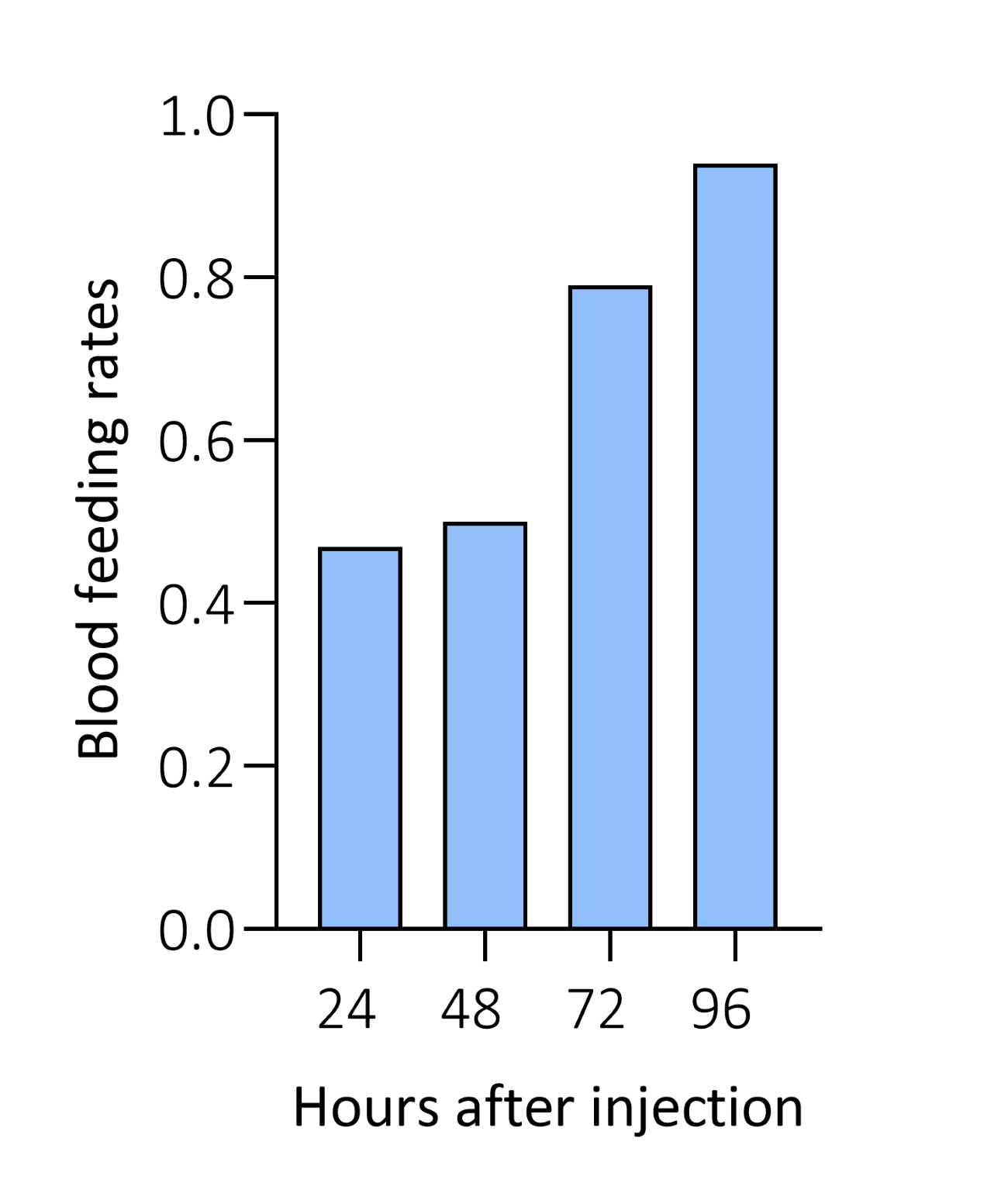

Supplement: S3 Fig — Bars represent the ratio of the percentage of blood-fed PBS injected mosquitoes over the percentage of blood-fed non-injected control mosquitoes with 20–27 mosquitoes within each group in one experiment. (TIF) [file pntd.0013848.s003.tif]

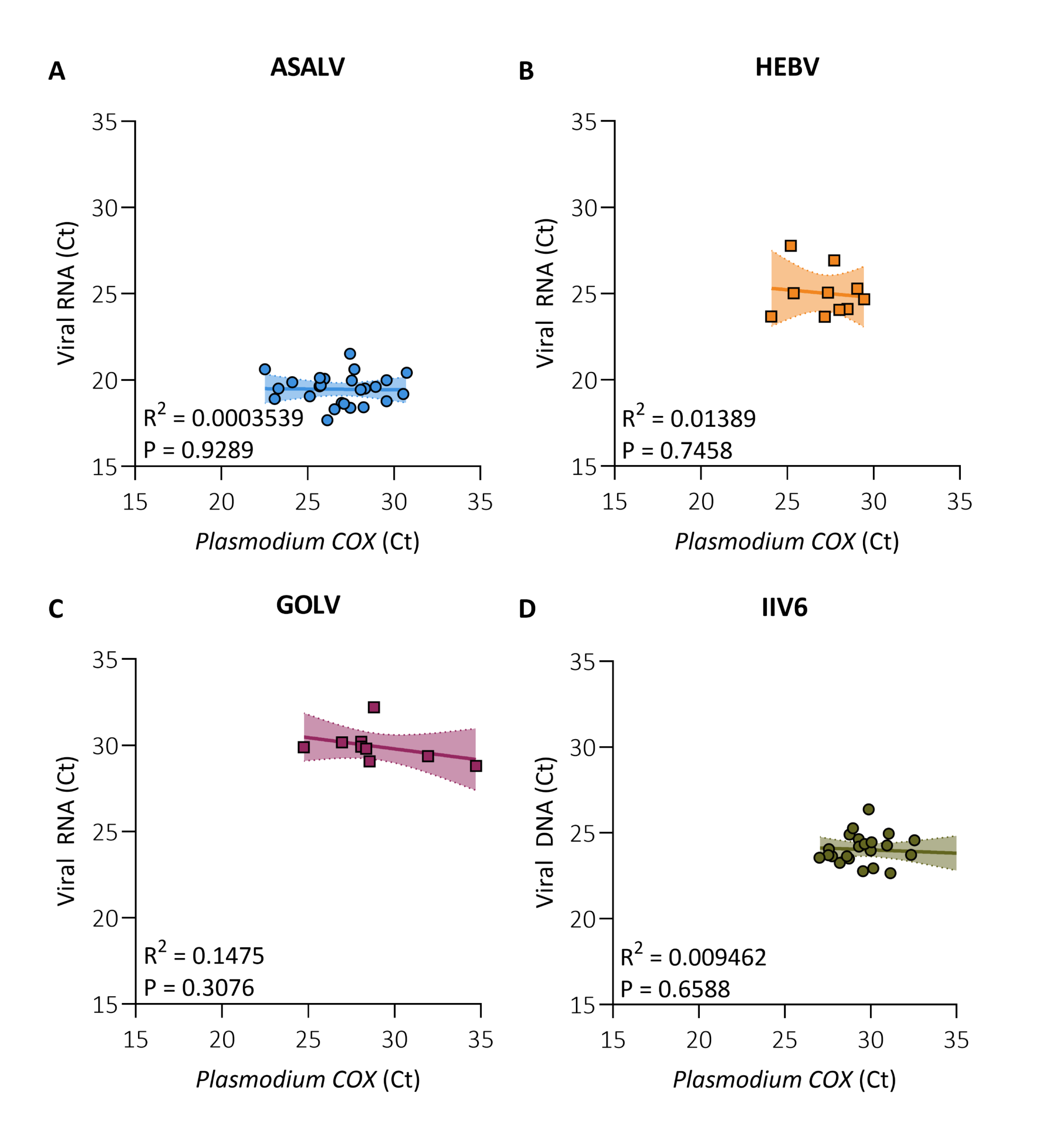

Supplement: S4 Fig — Each datapoint represents one virus and Plasmodium coinfected mosquito, with the Plasmodium (COX1) Ct value on the x-axis and corresponding viral Ct value on the y-axis for (A) ASALV, (B) HEBV, (C) GOLV and (D) IIV6. Linear regression lines were fitted, and Pearson R2 and P values are indicated. (TIF) [file pntd.0013848.s004.tif]
