# Supplementary material for: Systemic infection with insect-specific viruses does not affect Plasmodium sporozoite formation in Anopheles mosquitoes
Source: PLoS Negl Trop Dis. 2025 Dec 26;19(12):e0013848. doi: 10.1371/journal.pntd.0013848 (PMC12768362; doi:10.1371/journal.pntd.0013848)
Supplement: S1 Table — (PDF) [file pntd.0013848.s005.pdf]

Supplementary table 1: Primers used in this study

Description	Forward primer	Reverse primer
**cell fusing agent virus**	3'-GCAGCGGCGCTTTTGTGTGG-'5	3'-GCACTGCAAGGCATCCTCAC-'5
**Kamiti river virus**	3'-ATCCACAGCTGTAGGCCTTG-'5	3'-CAACCCGTCCGTTTGGTTTC-'5
**Culex Y virus**	3'-GCACTACTTGAGCACTCGGT-'5	3'-GGTAGTATTTGTCCGGCCCC-'5
**cricket paralysis virus**	3'-ACGAGGAAGCAACTCAAGGA-'5	3'-GAGCCCGCTGAGATGTAAAG-'5
**Flock House virus**	3'-GTTTAATCGGACCGAAGTGC-'5	3'-TATTCCGGCTTCTACGTTGG-'5
**invertebrate iridescent virus 6**	3'-ACACCTATCATGGGAACTTGGG-'5	3'-GGACCCGTTTACCTTCATTGTACG-'5
**La Crosse orthobunyavirus**	3'-GGCATGATTGCAGGCCTAA-'5	3'-TCTGTCTGAAGCCTGACCTG-'5
**Ferak orthoferavirus**	3'-GCATTGTTTAGCCTGTGTGCT-'5	3'-ATCAACCCTGTGTGGGTGAG-'5
**Jonchet orthojonvirus**	3'-AAAGCGACACTAGCGGTTCT-'5	3'-AAGGGCCTTCATTCCCAACC-'5
**Gouléako goukovirus**	3'-AGCCCAAACAACTAGCGGAG-'5	3'-CCTCCAGGGAGCAAGATTGG-'5
**Herbert herbevirus**	3'-CGTGATTGTGATGCACAGGT-'5	3'-GGCCCAGCTAAGTCAACCTT-'5
**Wallerfield virus**	3'-ATTTGGGAGTGCCAGATTAC-'5	3'-ATACGTAGCGGCACTTCCTG-'5
**Piura virus**	3'-TTGGTTCAACGCACACGTTC-'5	3'-ACCACAGCCATCAGGAACAG-'5
**goutanap virus**	3'-TTCGTATACCTCGGCGTACC-'5	3'-TTGCAGCACTTCCAGATTCC-'5
**Agua Salud virus**	3'-CTCGAAGAGAGGCTCACCAC-'5	3'-CGCAGAAGTAAGGCGGTTTG-'5
**Cavally virus**	3'-CAGCAATACCATTGCGCGAC-'5	3'-TCCATGTCGGAGTAGTGCTG-'5
**Otulum virus**	3'-GATGCCAACGTAAACCTAGC-'5	3'-ATGTGCCAAAATCGTAAGCG-'5
**Panama sandfly flavivirus**	3'-CATGGATGACCGCTTTGCTG-'5	3'-TGGTTCGAGCAGAAGGGAAC-'5
**Agua Salud negevirus**	3'-TAACACGTGCTTTTGTGCGG-'5	3'-TGACCGGATCGGGTAAAAAC-'5
***Anopheles* RPL5**	3'-TCGGCCTGCACGTCGCCAACTACA-'5	3'-GGGATCCTGATGGAGGCGTGGGCG-'5
***An. gambiae* Cecropin 1**	3'-TCCAAGATCTTCATCTTTGTCG -'5	3'-CTTAACGCCTGCCACCAC-'5
***An. gambiae* Defensin 1**	3'-TACCCTTCTGGACGAACTGC -'5	3'-CACACACCGCCTTACTGTTG -'5
***An. gambiae* Gambicin 1**	3'-TGAAGCAGGTGTGCATTCTT-'5	3'-AACTTTCGCTTGCAGTCCTC-'5
***Plasmodium* 18S rRNA**	3'-TCCGATAACGAACGAGATCTTAAC-'5	3'-ATGTATAGTTACCTATGTTCAATTTCA-'5
***Plasmodium* COX1**	3'-CATCAGGAATGTTATTGCTAACAC-'5	3'-CGATCTCCTGCAAATGTTGGGTC-'5
